# MIA - A free and open source software for gray scale medical image analysis

**DOI:** 10.1186/1751-0473-8-20

**Published:** 2013-10-11

**Authors:** Gert Wollny, Peter Kellman, María-Jesus Ledesma-Carbayo, Matthew M Skinner, Jean-Jaques Hublin, Thomas Hierl

**Affiliations:** 1 Biomedical Imaging Technologies, ETSI Telecomunicación, Universidad Politécnica de Madrid, Ciudad Universitaria s/n, Madrid 28040, Spain; 2 Human Evolution, Max-Planck-Institute for Evolutionary Anthropology, Deutscher Platz 6, Leipzig, Germany; 3 Ciber BBN, Zaragoza, Spain; 4 UCL Anthropology, Gower Street, London, UK; 5 Laboratory of Cardiac Energetics, National Heart, Lung and Blood Institute, National Institutes of Health, DHHS, Bethesda, MD, USA; 6 Department of Oral and Maxillo Facial Plastic Surgery, University of Leipzig Liebigstr. 10-14, Leipzig 4103, Germany

## Abstract

**Background:**

Gray scale images make the bulk of data in bio-medical image analysis, and hence, the main focus of many image processing tasks lies in the processing of these monochrome images. With ever improving acquisition devices, spatial and temporal image resolution increases, and data sets become very large.

Various image processing frameworks exists that make the development of new algorithms easy by using high level programming languages or visual programming. These frameworks are also accessable to researchers that have no background or little in software development because they take care of otherwise complex tasks. Specifically, the management of working memory is taken care of automatically, usually at the price of requiring more it. As a result, processing large data sets with these tools becomes increasingly difficult on work station class computers.

One alternative to using these high level processing tools is the development of new algorithms in a languages like C++, that gives the developer full control over how memory is handled, but the resulting workflow for the prototyping of new algorithms is rather time intensive, and also not appropriate for a researcher with little or no knowledge in software development.

Another alternative is in using command line tools that run image processing tasks, use the hard disk to store intermediate results, and provide automation by using shell scripts. Although not as convenient as, e.g. visual programming, this approach is still accessable to researchers without a background in computer science. However, only few tools exist that provide this kind of processing interface, they are usually quite task specific, and don’t provide an clear approach when one wants to shape a new command line tool from a prototype shell script.

**Results:**

The proposed framework, MIA, provides a combination of command line tools, plug-ins, and libraries that make it possible to run image processing tasks interactively in a command shell and to prototype by using the according shell scripting language. Since the hard disk becomes the temporal storage memory management is usually a non-issue in the prototyping phase. By using string-based descriptions for filters, optimizers, and the likes, the transition from shell scripts to full fledged programs implemented in C++ is also made easy. In addition, its design based on atomic plug-ins and single tasks command line tools makes it easy to extend MIA, usually without the requirement to touch or recompile existing code.

**Conclusion:**

In this article, we describe the general design of MIA, a general purpouse framework for gray scale image processing. We demonstrated the applicability of the software with example applications from three different research scenarios, namely motion compensation in myocardial perfusion imaging, the processing of high resolution image data that arises in virtual anthropology, and retrospective analysis of treatment outcome in orthognathic surgery. With MIA prototyping algorithms by using shell scripts that combine small, single-task command line tools is a viable alternative to the use of high level languages, an approach that is especially useful when large data sets need to be processed.

## Background

Imaging modalities like *ultrasound*, *magnetic resonance imaging* (MRI), *computed tomography* (CT), *positron emission tomography* (PET) make out the bulk of images used in bio-medical imaging and image processing. Common to these imaging modalities is that they all provide gray scale images, and although image fusion steadily gains importance in image analysis, the focus is still on processing these gray scale image, albeit only to properly fuse modalities.

Challenges arise from the ever increasing improvement of the imaging devices. On one hand, the spatial resolution is increasing. For instance, in anthropological research high resolution data sets of teeth that are obtained by using *micro CT* (*μ*CT) can easily reach 20GB. Likewise, in medical research 7-Tesla MR scanners that offer imaging at high resolution with a reasonable acquisition time are more and more used and will most likely soon find their way into the clinical routine. On the other hand, the temporal resolution of data acquisition increases making it possible to monitor dynamic processes, like the beating heart, in real time, which also results in large data sets. As a result, the software that is used to process this data must handle working memory efficiently.

Image processing frameworks exist that focus on easy prototyping, like SciLab [1], ImageJ [2], ICY [3], or the image processing toolbox in Matlab. These frameworks make it easy to develop new algorithms also for scientists who have little or no experience in software development. One of the reasons why these frameworks are easy to use is that they hide the difficulties of memory management, i.e. how and when working memory is allocated, reused, and released. Without this kind of control the developer can not specify when memory is to be released, instead this is taken care of by, e.g., a garbage collector (cf. e.g. [4]) that usually works asynchronously. In addition, researchers inexperienced in structured and object oriented programming tend to write code where temporary variables are kept alive, even though they are no longer used. Hence, when running algorithms that allocate memory temporarily, the amount of working memory that is actually used may vastly exceed the amount of memory that the algorithm theoretically requires. When working with large data sets an image processing task may easily require more memory that is installed in the work station, resulting in the corresponding program being terminated by the operating system before it can finish processing the data. This makes it difficult to implement algorithms that are supposed to work on large data sets with languages that use this kind of automatic memory management.

To avoid these problems when developing new algorithms, one can try to prototype by working with small example data sets, and then translate the prototyped algorithm to an implementation that gives the developer full control over memory handling. However, switching from one programming language used in prototyping to another in the final implementation puts an additional burden on the developers and introduces an additional source for errors.

As a second alternative one can also prototype directly in a language that gives full control over memory management like C or C++, where the programmer can explicitly decide when to allocate and free memory chunks. Here, OpenCV [5] that focuses on computer vision tasks in 2D images, and the *insight toolkit* (ITK) [6] could be used as a basis. Especially the latter is very well suited for bio-medical image processing. It is provided under a permissive license, comes with an in-depth documentation, is backed by a commercial company, Kitware, and enjoys a large user base. However, various reasons exist why an alternative to prototyping in C++ by using, e.g., ITK might be sought for: Prototyping on a *change code - compile - run* basis is very time consuming because compiling C++ code is notoriously slow [7], especially when dealing with heavily templated code (like in ITK) that requires that the implementation of most algorithms is provided by including C++ header files that need to be processed in each compilation run, even though they do not change when the prototype algorithm implemented in the main compilation unit changes. In addition, developing in C++ is quite challenging for researchers inexperienced in software development.

A third option for algorithmic prototyping is to use a combination of command line tools and a scripting language. Here, the hard disk becomes the temporary storage - a figuratively limitless resource when compared to the available random access memory (RAM) of a workstation. A command line tool based prototyping has also the advantage that the researcher doesn’t need to know software development, only a basic understanding of a shell scripting language is required to facilitate some automation when developing new algorithms.

Toolkits that support this kind of command line based programming are e.g. the FMRIB Software Library (FSL) [8] and Lipsia [9] that are specifically designed for the analysis of 3D FMRI, MRI and DTI brain imaging data. Other software tools that may be of interest in this context are elastix [10] and NiftyReg [11] that focus on image registration only, and NiftySeg [12] that is tailored for very specific segmentation tasks. Because of their specific focus, these software packages are not very well suited as a basis for a general purpose gray scale image processing. With ITKTools [13] a set of image processing command line tools exists that expose ITK functionality, but their documentation is somewhat limited. In addition, above toolkits don’t define a clear road for the transition from the script based prototype to a stand alone program.

Other software for image analysis exist that go beyond image processing, even provide graphical user interfaces and the means for visualization, e.g. 3D Slicer [14], the Medical Imaging Toolkit (MITK) [15], and MeVisLab [16]. Out of these, only 3D Slicer is free software, i.e. its source code is available and allows redistribution. 3D Slicer is a platform that provides many means for image processing and visualization, and acts as a front end to a variety of image processing back-ends, to which it interfaces either by direct linking of libraries, through plug-ins, or command line calls to external programs. However, 3D Slicer also uses python scripting for algorithmic prototyping with the implications arising from automatic memory management described above.

Apart from these problems regarding specifics of the implementation and memory management, it is also of interest to provide alternatives to widely used implementations of algorithms in order to assure the reproducibility of research.

### 

**Program 1.** If the pixel type is neither known nor one specific pixel type required, the function **mia::filter** is used to dispatch the image processing to a functor that implements the filter for all available data types, usually by means of a template.

### Contribution

With MIA we provide a free and open source software package written in C++ that strives to solve some of these challenges: It can be used for prototyping new algorithms by working interactively on the command line and implement some automation by using a shell scripting language, which makes it also accessible to researchers that are not experienced in software development.

Because in this phase of algorithm development the hard disk is the temporal storage, memory requirements are generally a non-issue. For very large data sets MIA also provides an interface to run certain image processing tasks *out-of-core*, i.e. by only keeping a subset of the data in the working memory.

MIA also provides libraries that can be used to shape newly created algorithms into new command line tools. Because a string based interface is used to describe filters, optimizers, etc., the algorithmic description on the command line is often very similar to the implementation in C++ code, making a transition from the former to the latter easier.

Since the functionality of MIA is mostly provided by plug-ins and one-purpose command line tools, it can easily be extended without the need to touch original code. The reliability and trustworthiness of the library and plug-in code is ensured by unit-tests [17]. Finally, using C++ as programming language leaves memory management in the hands of the software developer, and hence, she can tune the algorithms accordingly when the limit of the available working memory becomes an issue.

In the following sections, the design and the development methodology of MIA are described in more detail. Examples are given how the software can be used interactively, how the transition from shell script to C++ program can be achieved, and options are discussed on how the software can be extended for ones own needs. Finally, examples of the successful application of the software in image analysis tasks are discussed and remarks about the current state and future directions conclude the article.

## Implementation

**Figure 1:**
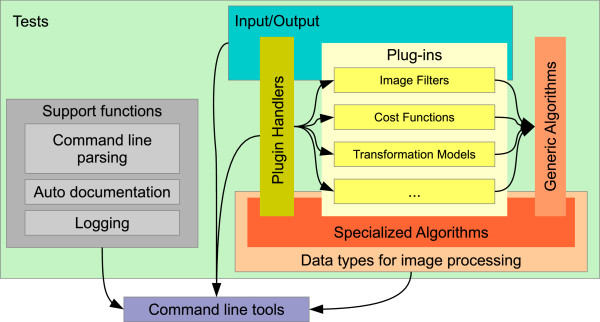
**The logical structure of MIA.** Library functions and plug-ins are under test to ensure the reliability of the implementation. Command line tools are provided as logically independent entities that are either only wrappers around library functions or implement complex tasks that require extra validation.

MIA is a general purpose image processing toolbox written in C++ that puts its focus on gray scale image processing. It consists of the following main building blocks as outlined in Figure 1: 

1. data types for image processing,

2. specific, task oriented algorithms,

3. plug-ins that provide specific functionality,

4. generic algorithms whose specific workings are defined at run-time by indicating the appropriate plug-ins,

5. tests to ensure the correct implementation of most of above functionality,

6. logging facilities,

7. a command line parser that provide the means for the automatic creation of the documentation for command line tools, and

8. command line tools for interactively running image processing tasks.

In addition to these components that directly form part of the MIA software, we also provide some add-on tools which can be used for the visualization of results and basic manual segmentation tasks.

The functionality of MIA is split into a core library for basic infrastructure, libraries dedicated to 2D and 3D image processing, and a library for very basic 3D mesh handling. The generic image processing functionality provided includes image registration, image filtering and combination, tools for the creation of synthetic images and transformations, and image segmentation. In order to reduce duplication of basic functionality that is available elsewhere, MIA makes use of external libraries for image in- and output, optimization, Fourier and wavelet transforms, independent component analysis, and unit testing (cf. Table 1). In a few cases, freely available code was directly incorporated into the library [18,19].

**Table 1:** Required software packages

**Package**	**Additional information**
CMake (≥ 2.8)	http://www.cmake.org
C/C++ compiler	ANSI compatibility and support for some features of the C++11 standard [35,36] are required. - GNU g++ (≥ 4.6) http://gcc.gnu.org and clang (≥ 3.2) http://clang.llvm.org are known to work.
GIT (≥ 1.7)	GIT - the fast version control system http://git-scm.com/ to download and manage the source code.
BOOST (≥ 1.46.1)	The BOOST library http://www.boost.org
CBLAS	e.g. ATLAS http://math-atlas.sourceforge.net
GSL (≥ 1.14)	The GNU Scientific Library (GSL) http://www.gnu.org/software/gsl
Intel TBB (≥ 3.0)	Intel threading building blocks for open source http://threadingbuildingblocks.org
libxml++ (≥ 2.34)	The C++ wrapper for the libxml XML parser library libxml++ and all its dependencies, http://libxmlplusplus.sourceforge.net
fftw (≥ 3.0)	Fast fourier transformation http://www.fftw.org

In the following, the building blocks of MIA are discussed in more detail, we will give example code fragments to illustrate some of the inner workings of the software, and we briefly discuss the add-on tools.

### Data types for image processing

MIA focuses on gray scale image processing, and therefore, it supports images with the following pixel/voxel types: Boolean for binary images and masks, integer valued types (signed and unsigned) of 8, 16, and 32 bits, (on 64 bit platforms 64 bit integers are also supported), as well as floating point valued pixels (single and double precision).

**Figure 2:**
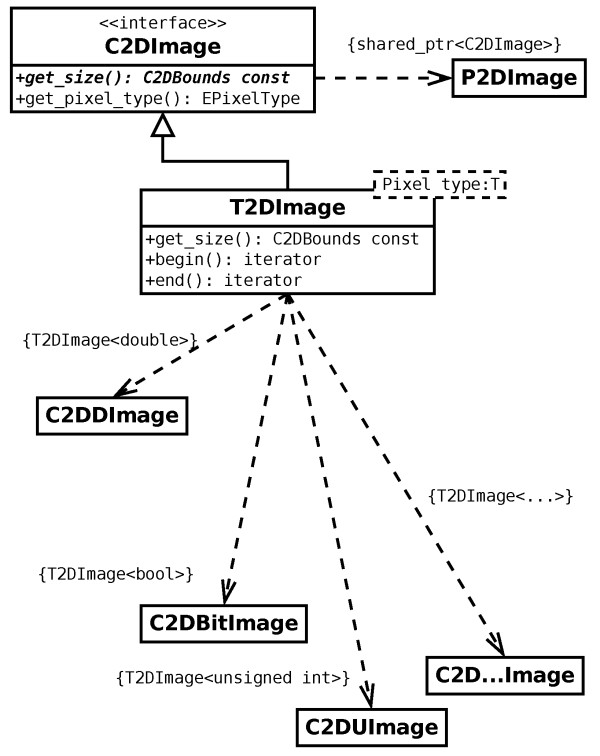
**The images are passed around in the code either as references to the abstract base class C2DImage or the corresponding shared pointer.** The actual image data is held within the derived templated class and the resolution of the actual pixel type is only done when the pixel data needs to be processed.

Currently, MIA supports the processing of 2D and 3D images, and of series of such images. In order to handle the different pixel types in a way that is mostly transparent to the application developer, a class hierarchy (Figure 2) is used that employs an abstract base class C2DImage (the 3d implementation uses an equivalent notation by replacing "2D" by "3D"), and its (shared) pointer type P2DImage. This abstract base class holds only meta-data but no pixel data and it provides the interface to query this meta-data including image size and pixel type.

The actual images make used of the class T2DImage which is a child class of C2DImage and templated over the pixel type. Hence, within the code the image data is passed around by means of the abstract base class and its respective (shared) pointer type, and only for image processing the actual pixel type needs to be resolved.

The preferred approach to resolve the specific pixel type of an image is a callback mechanism that implements the processing of all possible pixel types by means of a templated functor (Program 1). This approach can also be used to provide pixel type specific code paths by using (partial) template specialization (cf. [20]).

In cases where an algorithm only supports one pixel type, or the actual pixel type is known because of the preceding processing, dynamic casting can also be used to resolve the pixel type.

The templated image types (as well as other data containers) that are implemented in MIA are compatible with the *C++ standard template library* (STL) [21] containers, i.e. they provide the required iterators, access functions, and type traits to make the application of algorithms provided by the STL and compatible libraries (e.g. BOOST [22]) possible.

### Filters and pipelines

Most image processing algorithms require the execution of series of filters that are usually chained, to form a pipeline, and of course, MIA supports this kind of processing from the command line and within the C++ code.

In MIA, filters act as functions, i.e. after initialization parameters can not be changed. Filters are only implicitly chained together to form a filtering pipeline based on their ordering in the appropriate command or function specification. Within the application each filter is called in turn, the obtained result overwrites the original data and is then passed to the next filter to simulate a pipeline.

To filter images MIA provides two modes of processing: The basic 2D and 3D filter that process complete images one at a time, and the *first-in first-out filter* (FIFOF) described in more detail in the next section that makes it possible to interpret a series of n-D images as one (n+1) image.

Given the comparable small memory footprint of the filter objects themselves, the memory requirement for a given filter chain composed of basic filters is effectively independent of the filter chain length.

**Figure 3:**
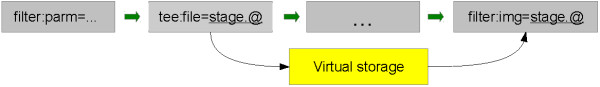
**Construction of non-linear pipelines by using the****tee****filter and virtual storage.** Note, the virtual storage is located in main memory.

To create filter chains that are not linear, i.e. chains where results of early filtering stages are used as input in later filter stages, a virtual storage system is implemented (Figure 3) that holds the data in working memory. By adding, e.g. the tee filter at the appropriate pipeline position an intermediate result can be stored in the virtual storage from where it can be retrieved later.

#### 

**Program 2.** Invocation of a non-linear registration of two images moving.png and fixed.png by using (1) a spline based transformation model with a grid spacing of 5 pixels and penalizing the magnitude of the separate norms of the second derivative of each of the deformation components [23], (2) minimizing the sum of squared differences, (3) by using L-BFGS for minimization with a maximum of 200 iterations, and stopping if the absolute change of all transformation parameters is below 0.001, and (4) writing the result to registered.png.

### Out-of-core processing

In order to process data sets that would require such a large amount of memory that they do not fit in the available working memory, in addition to the basic filter infrastructure given above, an alternative filter infrastructure is provided that interprets a series of n-dimensional images as one (n+1) dimensional image and, hence, makes it possible to process series of n-D images as if it were one (n+1)-D image.

Two types of processing may be considered: accumulation and filtering. Accumulation can be implemented straightforward, since it only requires to load each image slice of a set, and accumulate the requested quantity, e.g. a histogram. Filtering, on the other hand is implemented similar to a *first-in first-out* buffer that we call a *first-in first-out filter* (FIFOF). This approach is - on a very basic level - comparable to the ITK streaming API; one notable difference is that with ITK the input image may actually be one large file and the number of data chunks is defined by the program, whereas with MIA, the input is always given as a series of files and the number of data chunks corresponds to the number of input files.

Like with basic filters these filters can be chained together, so that different filters can be run one after another, without writing intermediate results to the hard disk. The last FIFOF in the chain usually implements the disk write operation. Apart from the chaining operation, of the two operations a FIFOF makes visible to the user one provides the means to push an image slice into the FIFOF chain and the other to finalize the filtering. Each FIFOF itself contains a buffer to accumulate and process slice data according to the implemented filter. In the simplest case, this buffer is used to store the number of n-D slices required to provide the (n+1)-D neighborhood information needed by the filter. In more advanced cases, the buffer may also store additional intermediate results.

During the course of the operation of a FIFOF. three stages can be distinguished: 

• First slices are pushed into the FIFOF, until the buffer contains the minimum number of n-D slices required to start (n+1)-D filtering. This push operation might already prepare the input data in some way. For example, in the (separable) Gaussian filter, a buffer stores slices that are already filtered in two dimensions.

• The buffer is then processed to obtain an output slice that is then pushed further down the chain. In addition, if the buffer has reached its maximum fill, the first slice is removed from the buffer, making room for the next input.

• When no more input is available, the finalize operation of the FIFOF is executed. It takes care to run the filtering of the remaining buffer content and to push its result down the filter chain.

With this filter structure, the requirement on working memory for a filter chain depends on the filters provided, and the size of an image slice of the image stack to be processed. Given an image slice size of *w* × *h*, and since the (n+1)-D filter width is usually small compared to the image size, the memory requirement of a filter chain can be expressed as *O*(*wh*) and, therefore, independent from the number of slices to be processed. However, this filter structure only provides the means to run filters that process the 3D data in one single pass. This rules out frequency domain filters as well as filters that need to be solved iteratively, like e.g., anisotropic diffusion.

#### 

**Program 3.** Bash script for the segmentation of the brain from a T1 MRI. The values ${...} are user provided parameters. It would also be possible to run each filter separately, and store the result for algorithmic fine-tuning without the need to re-do the whole pipeline.

Note, however, although the basic infrastructure for this type of processing is independent of the dimension, currently only filters for the processing of a series of 2D image as a single 3D image are implemented.

### Plug-ins and algorithms

In MIA, dynamically loadable plug-ins provide file in- and output for most supported data and image types, and the means to specialize generic algorithms. Plug-ins are modules that implement the specialization of a certain generalized interface and are loaded during the run-time of a program. Thereby, they provide an elegant way to extend the functionality of such a software without touching its original code base.

An example how to invoke various different plug-ins to specialize a registration algorithm by invoking the respective plug-ins (i.e. transformation model, the image similarity measure to be optimized, and which optimization method should be used) is given in Program 2.

Currently, the following functionality is provided by plug-ins: data file in- and output, image filters and combiners, image similarity measures, transformation models, function minimizers, neighborhood shapes, interpolation kernels, and interpolation boundary conditions (see also below, section *Overview over available algorithms and filters*). Each type of plug-in is managed by its own plug-in handler. To avoid superfluous hard disk access that would result from initializing the same plug-in handler multiple times, plug-in handlers are implemented using the singleton design pattern [24], i.e. only one plug-in handler exists for a plug-in type during the run-time of the program. When the plug-in handler is first invoked, the available plug-ins are loaded and the plug-in list can not be changed afterward, i.e. no additional plug-ins can be added during the run-time of the program. However, for command-line based programs this is usually not a problem.

### Command line parser with auto-documentation

Although various implementations of command line parsing libraries exist (e.g. *popt*[25], *BOOST program options*[26]), they lack the option of generating a cross referenced documentation of command line tools and available plug-ins. Therefore, the command line parsing was implemented from scratch to provide the means of directly generating objects (filters, cost functions,...) from their string based plug-in descriptions, and to generate an exhaustive description of the according command line tool in XML format that describes its command line options and the plug-ins that may be used by the tool. This output can then be used in two ways: The creation of documentation and introspection. Incorporated into the MIA build process is the creation of man pages and a cross referenced documentation in Docbook format [27] of the command line tools and plug-ins. The latter is also used to create a HTML based user reference, i.e. [28]. Since the XML descriptions of the command line tools and plug-ins provide the introspection that is needed to automatically form proper calls to MIA programs, they could also be used to automatically create interfaces to MIA functionality.

### Tests

In order to ensure reliability of the software, all plug-ins and most algorithms are implemented following the *test driven development* (TDD) paradigm [17]. BOOST test [29] is used as the testing framework. Since plug-ins provide a very specific functionality that can usually be tested easily, the build system strongly encourages the developer to provide the program that implements the according unit tests. For many algorithms the same assumption about testing can be made, and hence, unit tests are provided. However, for some complex algorithms, like e.g. non-linear image registration, or certain segmentation algorithms the expected result can not easily be defined, because they depend on many factors, like which optimization algorithm is used, which parameters are defined, and sometimes, algorithms even use a random component for initialization. In these cases, sensible unit-testing is impossible and the proof of correctness of the implementation requires a task specific validation. Currently, we estimate that approximately 65% of the library and the plug-in code is covered by unit tests.

#### 

**Program 4.** Sketch of a C++ program for the segmentation of the brain from a T1 MRI (The full code can be found in the file src/3dbrainextractT1.cc of the MIA source code distribtion.) The parameters *infile*, *outfile*, *wmprob*, *thresh*, and *growshape*, may be defined from the command line.

### Command line tools

The command line tools that are implemented in MIA provide the means to run image processing tasks without the need for software development. This makes MIA also usable for researchers with no or only a limited experience in software development.

These tools follow the Unix-philosophy *one tool, one task*, i.e. each command line program is designed to run exactly one type of image processing task. This task may be as simple as running a series of filters on an image, or combine two images. In these cases the tools are simple drivers to the library functionality that is already developed by using TDD, and it only needs to be ensured that the parameters are properly passed to the library functions. A command line tool may also comprise a complex task like running a motion compensation algorithm on a series of images. Here a full, task specific validation is required, for example, like it was done in [30-32] for various motion compensation algorithms.

Below, in section *Overview over available algorithms and filters* an overview of the available tools is given.

### Add-on tools

The add-on tools that are maintained in conjunction at the MIA web page http://mia.sourceforge.net consist of a software *miaviewit* that is used for basic visualization tasks, a library *mialm*, and a volume surface renderer *mialmpick* that can also be used to pick landmarks in 3D volume data sets, and *pymia* bindings that makes some of the functionality available to python and also provide a simple program for the manual segmentation of myocardial images.

## Using and extending MIA

**Figure 4:**
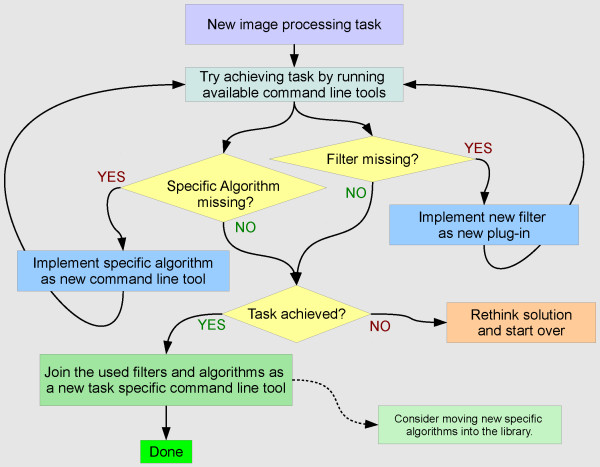
Suggested work-flow for tackling new image processing tasks.

The software can be utilized in various ways: The command line programs can be called to run certain image processing tasks ad-hoc, and one can make use of the library to create new programs that combine the available image processing operators. To tackle a certain image processing task, MIA is designed to accommodate a work-flow for the development of new algorithms as illustrated in Figure 4.

The command line programs provide a flexible means for algorithmic prototyping based on interactive execution of atomic image processing tasks and their combination in shell scripts. If functionality is missing, like e.g, a filter, image combiner, or some specific algorithm, the design that is based on the combination of plug-in and task specific command line tools makes it easy to add this functionality without touching existing code. Hence, only little development overhead is required in the process of prototyping, and no additional testing is required to ensure that new code does not break existing functionality.

Once a working prototype algorithm is implemented as a shell script, moving to a tool written in C++ is made easy, because the driving feature of most processing done by using MIA, that is, specifying how the functionality of certain plug-ins should be invoked, is very similar in both cases. For example when comparing how the image filters are specified in a shell script (Program 3) versus in C++ source code (Program 4), one can see that the filter descriptions are essentially the same, only the (language specific) glue code changes.

### Image processing on the command line

As an example on how the command line tools can be used to achieve a certain image processing task, a simple algorithm to peel the skull and skin from a T1 magnetic resonance image (MRI) will hold forth.

The tools are applied as given in Program 3 with the following objective: First, a 3-class adaptive fuzzy c-means segmentation is run to correct the *B*^0^ gain field [33], and to obtain a segmentation of the image into background, white matter, and gray matter (line 1). Then, the probability image related to the white matter is selected (line 2). Finally, the following filter chain is run to obtain the masked brain (lines 3–6): An initial white matter mask is extracted by binarizing the probability image, the mask is shrunk and small connections are eliminated by running a morphological erosion. Then connected components are labeled and the largest connected component is selected as the one representing an approximation of the white brain matter. This approximation of the white matter is used to initialize a region growing algorithm on the *B*^0^ field corrected image using a given neighborhood shape and intensity threshold to stop region growing. Alternatively, region growing is stopped, if all neighboring pixels that are not yet labeled have a higher intensity value than the according seed pixel. Then, morphological closing and opening are run to eliminate small holes and smooth the mask boundary, and finally, the obtained mask is used to extract the brain from the *B*^0^ field corrected image. The parameters *infile*, *outfile*, *wmprob*, *erode_shape*, *thresh*, *growshape*, *close_shape*, and *open_shape* must be defined by the user in the shell environment.

For initial prototyping it may be better to run each filter separately, and store the result on the hard disk, in order to be able to tune the algorithm without re-applying the full filter chain. Note, that storing intermediate results to disk in itself imposes only a limited run-time penalty if the software is executed on a computer that has sufficient memory to hold all the data in working memory. Here, at least with Linux, the operating system will use this memory as disk cache and writes to the disk in the background without the program having to wait until the write operation is finished [34]. Hence, no run-time penalty is imposed on the software by the disk in- and output that goes beyond the operations that are needed to convert the data to and from the used in- and output file formats.

### Translating the shell script into a C++ command line tool

Given successful prototyping, one may then shape the obtained algorithm into a new command line tool. In the case of the example given above this is straight-forward and illustrated in Program 4. Here, lines 1–7 define the parameters that need to be set from the command line. Then, the command line options must be created and parsed (line 9–19).

After loading the image (line 31), the fuzzy c-means segmentation [33] with *B*^0^ gain field correction is run (line 22–24). In line 25–26, the gain field corrected image is used in the internal data pool to make it accessible for the plug-ins. Then, the white matter probability image is selected from the fuzzy segmentation class images (line 27). In the next step, the descriptions of the binarize (line 28–29) and region grow filters (line 30–32) are created. Finally, the filters are applied in the order given in the run_filters function call, to obtain the masked brain image from the white matter probability image and the gain field corrected image (lines 33–37) and the result is saved (line 38). Also note, the function run_filters is implemented as a *variadic template*[35,36] that can take an arbitrary number of parameters, and these parameters may be strings that described the filters as well as previously created filters.

In this example, the parameters *erode_shape*, *close_shape*, and *open_shape* from the shell script example above have been replaced by fixed values. Also note, that the specification of filters is very similar in shell scripts and C++ programs: In both cases, the filters are specified by string literals or constructed strings. All functionality in MIA that is provided by plug-ins is invoked by similar string based descriptions.

### Extending the library

The library may be extended in various ways: Firstly, one may add a plug-in to provide an additional specialization to an already implemented generic concept, such as adding a new cost function, filter, or optimizer. Considering the MIA code base, adding a new plug-in has the advantage of not being intrusive, i.e. the original code base will not be touched by doing so. An example on how a new plug-in is implemented can be found in the on-line documentation [37].

If new algorithms are to be added that do not fit into the abstract category provided by an existing plug-in type, the proposed approach is first to create a new command line tool that provides the intended functionality, and test it on appropriate data. Then, after proper testing, the algorithm may be moved to the core libraries as new functions and classes, leaving the command line tool as a skeleton program that handles the command line parsing and just calls the functions that were moved to the library.

Finally, specialized algorithms may be replaced by generic versions, moving functionality to plug-ins, and thereby making it interchangeable. In such a refactoring step, new generic concepts will be added that provide new plug-in types with their corresponding handlers and interfaces. This task is more intrusive since not only interfaces may be added to the library but also already available interfaces may be changed.

## Use cases

In this section the usability of MIA for image processing and analysis tasks will be illustrated by presenting three use cases: Motion compensation in myocardial perfusion imaging, out-of-core processing of high resolution data, and the evaluation of medical treatment. For the first two use cases the according data sets and scripts to run the analysis are available for download on the project web page. Since the data used in the third use case can not be completely anonymized (i.e. a 3D reconstruction makes an identification of the patient possible) it will only be made available on request. As an alternative for hands on experience with the software we provide the 3D data of a pig head that was surgically altered in two steps.

All experiments were run on a Gentoo Linux AMD64 workstation, facilitating an AMD Phenom II X6 1035T Processor, 16 GB of DDR3 RAM (1333 MHz); the software was compiled using GNU g++ 4.6.3 and the compiler flags were set to "*-O2 -g -funroll-loops -ftree-vectorize -march=native -mtune=native -std=c++0x*".

### Motion compensation in myocardial perfusion imaging

Perfusion quantification by using first-pass gadolinium-enhanced myocardial perfusion *magnetic resonance imaging* (MRI) has proved to be a reliable tool for the diagnosis of coronary artery disease that leads to reduced blood flow to the myocardium. A typical imaging protocol usually acquires images for 60 seconds to cover the complete first pass and to include some pre-contrast baseline images. To quantify the blood flow, the image intensity in the myocardium is tracked over time (cf. [38,39]).

In order to perform an automatic assessment of the intensity change over time, no movement should occur between images taken at different times. *Electrocardiogram* (ECG) triggering is used to ensure that the heart is always imaged at the same cardiac phase. However, since the 60 seconds acquisition time span is too long for average people to hold their breath, breathing movement is usually present in the image series.

Various methods for automatic motion compensation based on linear and non-linear registration have been implemented in MIA [30-32,40,41]. By implementing these methods all in the same general software framework, a fair comparison has been made possible, since differentiating factors like the use of different programming languages, different implementations of optimizations algorithms, etc. could be eliminated (cf. [32]).

**Figure 5:**
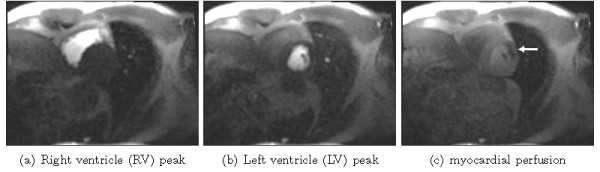
**Images from a first-pass gadolinium-enhanced myocardial perfusion MRI study, here taken at the moment when the contrast agent first enters the right ventricle (RV) (a), then the left ventricle (LV) (b), and finally, perfuses the LV myocardium (c).** Note, the hypointense region in the perfused myocardium **(c)** indicates a reduction in blood flow.

As an example consider the motion compensation applied to a perfusion data set of a patient considered clinically to have a stress perfusion defect that was acquired free breathing. First-pass contrast-enhanced myocardial perfusion imaging data sets were acquired and processed for one subjects under clinical research protocols approved by the Institutional Review Boards of the National Heart, Lung, and Blood Institute and Suburban Hospital. The patients provided written informed consent, and the analysis was approved by the NIH Office of Human Subject Research. Images were taken in 60 time steps for three slices (at the base, mid, and apical level). The first two time steps comprise proton density weighted images that may be used for intensity inhomogeneity correction (see, e.g., [42]); however, this intensity correction is not considered here. The remaining slices were acquired by using the SR-FLASH protocol. An example of the different time steps at the base level is given in Figure 5.

Motion compensation was achieved by using the ICA based method described in [32], running motion compensation like given in Program 5. The run-time of the motion compensation for one slice consisting of 60 frames took approximately 85s when the registrations were run serially, and 25s when run parallelized utilizing the six available processor cores.

#### 

**Program 5.** Run the slice wise motion compensation for a perfusion set within the directory where the original files are stored as dcm (DICOM) files. First, the slices are sorted into time series sets based on the recorded z-location. Then for each set ICA based motion compensation is run.

**Figure 6:**
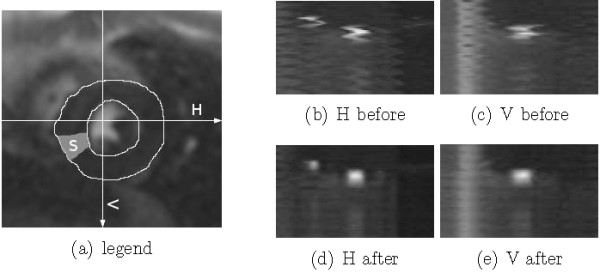
**Profiles obtained by cutting through the time stack at the locations indicated (a) before and after registration.** **(a)** Location of the horizontal (H) and vertical (V) cuts used to obtain the intensity profiles shown in **(b-e)**, as well as the location of the myocardial section (S) whose time-intensity profiles are shown in Figure 7. In the profiles cut through the time stack of the original series **(b,c)**, the breathing movement is clearly visible. In the registered series **(d,e)**, this movement has been considerably reduced.

**Figure 7:**
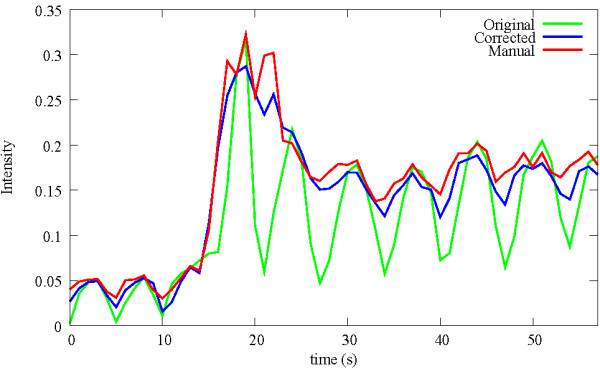
**Automatically obtained time-intensity curves before (green), and after (blue) motion compensation of the section S of the myocardium as indicated in Figure**6**(a), compared to the manually (red) obtained one.** Note, before motion compensation the oscillation of the automatically obtained curve (green) hinders an automatic analysis of the contrast agent uptake of the myocardium which is used to quantify blood flow. After motion compensation, the automatically obtained time-intensity curve (blue) follows closely the manual obtained one (red), making a proper quantification possible.

In order to qualitatively assess the success of the motion compensation, horizontal and vertical cuts (Figure 6 (a)) through the temporal stack of the slices can be visualized (Figure 6(b-e)), and a quantitative validation can be obtained by comparing automatically obtained time-intensity curves of the myocardium to manually obtained ones (Figure 7). It is clearly visible how the motion was eliminated from the image series, making an automatic analysis feasible. For a detailed discussion of this analysis and the validation of the according methods implemented in MIA by using a larger set of patients the reader is referred to [30-32]. Revised pre-prints of these articles are also available as downloads on the MIA project home page [37].

### Segmentation of high resolution *μ*CT data by out-of-core processing

Paleoanthropological research increasingly employs the use of non-destructive imaging technologies, such as microtomography. These technologies introduce several advantages into the research process, most importantly the preservation of valuable fossil and extant biological tissues (in lieu of physical and chemical alteration of these specimens to examine their internal properties). One research area where many samples are available is the analysis of teeth. Teeth dominate the fossil record, because of their resistance to diagenetic alteration and other degenerative taphonomic processes, and the ability to extract new information from their internal structures allows us to address important aspects of human evolutionary history such as processes of tooth development [43-45], species identification and diversity [46,47], and the evolution of human/primate diet [48].

**Figure 8:**
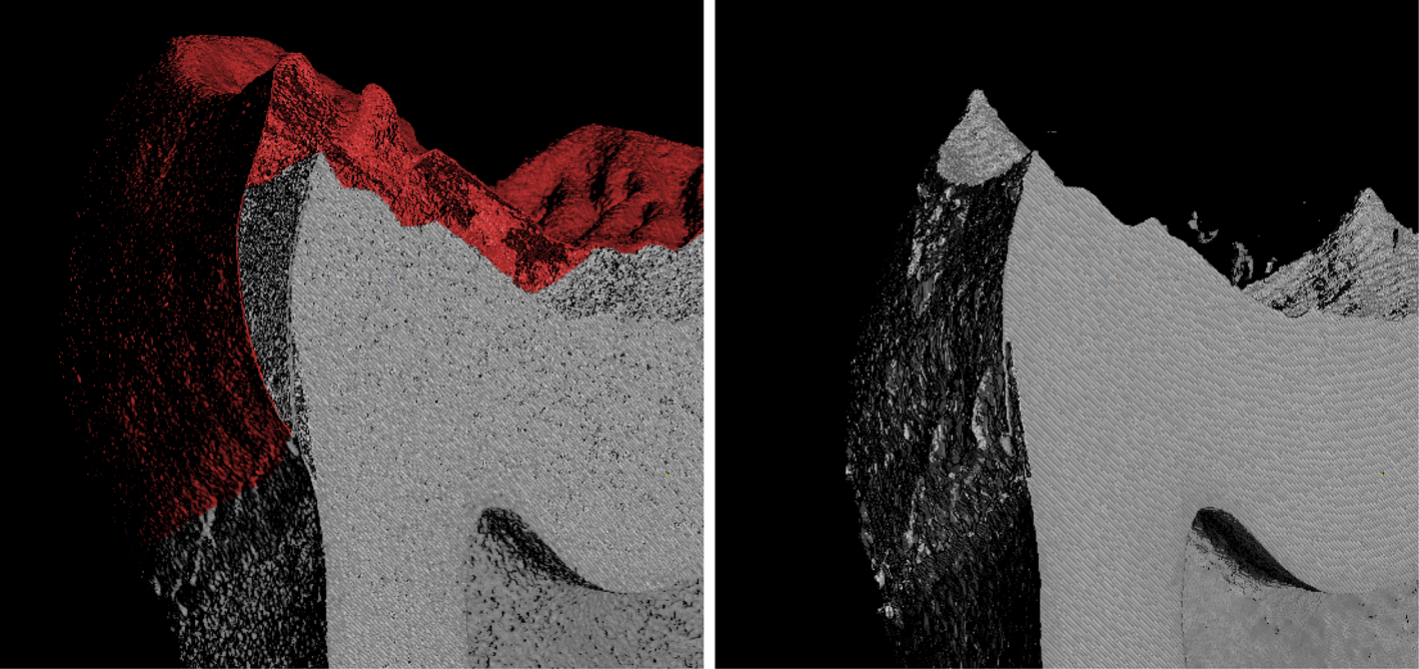
**Visualization of of a cut through the original (left) and filtered tooth data (right) by using *mialmpick *.** The left image was post-processed to colorize the offending pixels. All pixels with intensities outside the range [46,90] were discarded, the resulting surface is expected to correspond to the dentine. Before filtering the level of noise results in overlapping intensities for dentine and enamel at the air-enamel boundary that is not eliminated by this segmentation approach and results in the appearance of a dentine-like surface (colored red) mimicking the enamel cap (left). Running the out-of-core filters significantly reduces this overlap providing a better representation of the dentine surface (right).

Amongst others, the comparative analysis of tooth shape has applications for understanding the function of teeth as well as reconstructing the taxonomy and phylogeny of living and extinct mammalian species. High-resolution computed tomography has made possible accurate 3D digital reconstruction of both external tooth shape and internal tooth structure. However, since the image data is acquired at a high resolution (50 pixels per mm and more) the resulting data sets are very large (possibly up to 20 GB per tooth). Processing this data on workstation class computers can hardly be done using software implementations that require loading all data into the working memory, and do not provide the user with a tight control over the memory management, i.e. out-of-core processing is a requirement. Furthermore, the segmentation of teeth is particularly difficult to automate given the large number of interfaces between tissues (e.g., air-enamel, enamel-dentine, dentine-pulp, dentine-air, and dentine-bone). In particular, voxels at the air-enamel interface tend to overlap in gray scale value with dentine. This may result in a segmentation that falsely indicates the presence of dentine on the surface of the enamel cap (Figure 8 (left)).

**Figure 9:**
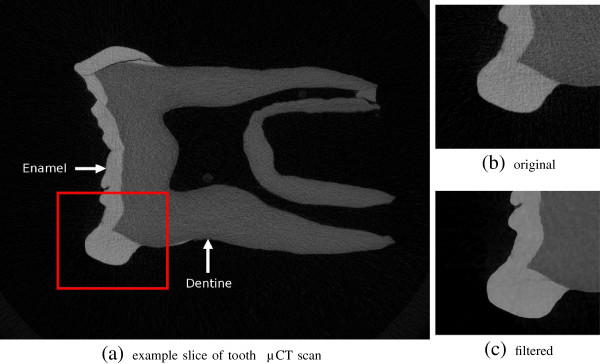
**Example slice of a primate molar scanned at a resolution of 0.028 × 0.028 × 0.028 ** ***mm***^**3**^** (a).** The original image **(b)** is very noisy making it difficult to properly segment the boundaries. After applying the out-of-core filtering, the tissue boundaries are more prominent **(c)** and segmentation can be achieved e.g. by thresholding.

The FIFO filtering implementation in MIA was specifically designed for this kind of data and as example of its applicability we illustrate here how the pre-filtering and segmentation of a high resolution microtomographic scan of a chimpanzee lower molar (isotropic voxel resolution of 0.028mm) can be achieved (Figure 9). The input data consisted of 769 slices at a resolution of 1824 ×1392 pixels (approximately 2 Giga-pixel). In order to obtain a segmentation of the enamel and dentine, first the images were smoothed with a median filter followed by the edge preserving mean-least-variance filter, both applied with a filter width parameter of two. These filters were applied as FIFO-filter to process the image series as a 3D volume. After filtering, an accurate automatic segmentation of dentine and enamel tissue can be easily achieved (Figure 8 (right)). Importantly, a proper segmentation reduces the necessity to edit by hand hundreds, or even thousands, of individual slices.

**Figure 10:**
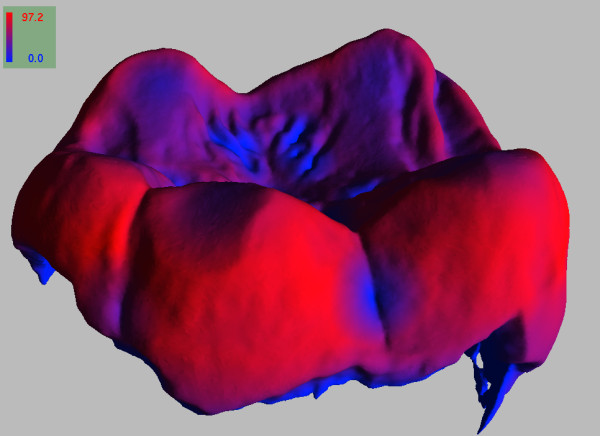
**Visualization the enamel thickness obtained by running the MIA tool chain on the provided example tooth data by using *viewitgui *.** The scale is given in pixel units, i.e. the maximum value of 97.2 corresponds to a thickness of approximately 2.7mm.

A segmentation then can either be achieved by using interactive tools, like e.g. Avizo, or by using additional filters available in MIA. In the case presented here, based on the all-over histogram of the images, a three-class fuzzy c-means classification of the intensity values was obtained, and enamel and background where segmented by using the classification to seed a region growing algorithm. Finally, the dentine was segmented as the remainder. For further analysis one may then evaluate the enamel-dentine boundary using morphological filters. By running a distance transform [49] on this boundary one can then, e.g. measure the enamel thickness that can give a variety of insights to human and primate evolution (cf. [48]). A visualization of the automatic evaluation of the enamel thickness of the example tooth is given in Figure 10.

The run-time to extract the enamel-dentine and the enamel air boundaries for the given data set was 90 minutes. Extracting the enamel surface, optimizing it by using a mesh-decimation algorithm implemented in the GNU Triangulated Surface library [50], and colorizing it according to the enamel thickness took another 60 minutes.

### Evaluating medical treatment

In many surgical specialties a certain need exists to evaluate treatment outcome. If pre- and post-therapeutic 3D datasets exist, MIA can be used to evaluate, quantify, and thus help to understand, therapy induced changes. This way MIA aids to improve treatment strategies and may improve future surgical outcome.

**Figure 11:**
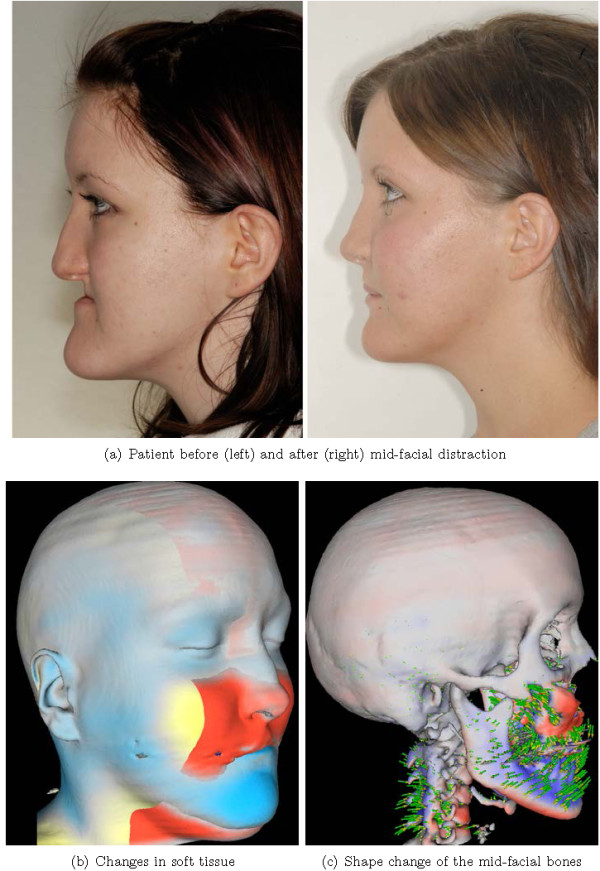
**A 17-year-old girl suffering from midfacial hypoplasia and retrognathia associated with isolated cleft palate (a, left).** Postoperative situation shows a harmonic maxillo-mandibular relationship and markedly improved esthetic appearance (**a**, right). Visualizing the changes by fluid-dynamical non-linear registration displays the complex changes caused by the forward-downward displacement and clockwise maxillary-mandibular rotation **(b, c)**. Red indicates displacements in the direction of the surface normal, and blue indicates displacements in the opposite direction of the surface normal. The visualization was obtained by using *miaviewit*.

As an example, we given the analysis of a treatment by means of orthognathic surgery (i.e. changing the position of the jaws). In this case, midfacial distraction osteogenesis using a rigid external distraction (RED) system was utilized to correct a midfacial hypoplasia and retrognathia associated with isolated cleft palate (Figure 11).

Using a RED system for midfacial distraction osteogenesis is a method to correct the underdevelopment of the midface, surpassing traditional orthognathic surgical approaches for these patients (e.g. [51]). In complex malformations, surgery planning is based on CT images followed by a modified midfacial osteotomy. Finally, the midface is slowly advanced by a halo-borne distraction device until a correction of the midfacial deficiency is achieved. Striking aesthetic improvements are obvious (Figure 11(a), left vs. right), but the analysis of the three-dimensional bony changes of the skull is necessary to get a better understanding of the effects of the distractor onto the whole skull, and thus, to improve therapy planning.

Based on the routinely acquired pre- and postoperative CT scans, and by using the fluid dynamics based image registration [52] implemented in MIA, the deformation field that described the underlying transformation could be estimated, and an analysis of the changes could be achieved (Figure 11(b,c)). Here the distraction osteogenesis resulted in a forward-downward displacement of the maxilla accompanied by a clockwise maxillary-mandibular rotation achieving the intended aesthetic improvements. The processing of these images of approximately 250^3^ voxels, including the rescaling to an isotropic voxel representation, rigid registration, non-linear fluid-dynamics based registration, and the extraction and optimization of the iso-surface that corresponds to the skull took approximately 15 min.

Based on this kind of analysis the outcome of the treatment by means of the RED system became more predictable resulting in better treatment outcome and higher patient satisfaction (cf. [53-55]).

For a landmark based validation of the method executed on 20 patient data sets the reader is referred to [53] (A pre-print of the article is also available on the MIA project home page [37]).

## Conclusion and future work

In this paper, we have presented a software package framework for general purpose gray scale image processing that is implemented in C++. Various image processing algorithms are implemented in MIA, amongst these specific segmentation algorithms, a variety of image filters and combiners, and generic image registration algorithms. One can make use of this functionality for ad-hoc image processing by running the various command line tools that are provided by the software. By offering an *application programming interface* (API) that exposes the specialized functionality provided by the plug-ins by the same string based interface that is used with the command line tools, the transition from script based prototyping to fully fledged programs is made easy. Because of its modular design that is based on dynamically loadable plug-ins and single task command line tools, adding new functionality to MIA is made easy and normally doesn’t require existing code to be changes, let alone recompiled.

We have illustrated the applicability of the software by providing examples from different research areas where the tool kit has and is being used. We showed how the software can be used for motion compensation of image series in the specific case of myocardial perfusion imaging, we presented an example of out-of-core image processing that is useful for the (pre-)processing of high resolution image data that is used in virtual anthropological research, and we provided an example for the retrospective analysis of mid-facial surgery by means of an external distraction device (RED) that has been and is used to improve the understanding of the underlying mechanics of the treatment.

The focus for the further development of MIA follows two main directions: On one hand, the code base is constantly improved by increasing test coverage, new algorithms are added as new application areas are explored. Here, of specific interest it is to improve existing and add new methods that are required for the processing and analysis of 3D+t data, and to introduce multi-threading to exploit the now commonly available multi-core hardware architectures, especially for the application of the software to high resolution and 3D data sets. On the other hand, given that the known user base of MIA users (i.e. of those who give feedback) is limited to the work groups of the contributing authors, it is in our interest to grow a lager community, and therefore, in order to encourage third party contributions, focus is also laid on providing tutorials for the use of and development with MIA.

## Availability and requirements

### Software

**Project name**: Medical Image Analysis

**Project home page**: http://mia.sourceforge.net/

**Operating system(s)**: POSIX compatible, Linux is tested.

**Programming language(s)**: C/C++

**Other requirements**: In order to compile the software the packages given in Table 1) are required. Additional functionality can be enabled if the packages given in Table 2 are also available.

**License**: GNU GPL v3 or later

**Any restrictions to use by non-academics**: None

**Table 2:** Supported external packages

**Package**	**Additional information**
DCMTK	DICOM image IO (partial support) http://dicom.offis.de/dcmtk
GTS	GNU triangulated surfaces library to support mesh processing and iso-surface extraction from volume data http://gts.sourceforge.net
IT++	Signal processing library http://itpp.sourceforge.net
NLopt	Nonlinear optimizers library http://ab-initio.mit.edu/wiki/index.php/NLopt
OpenEXR	A HDR image library that supports 32 bit integer and floating point valued images http://www.openexr.org
PNG	Portable network graphics http://www.libpng.org
TIFF	The tagged image file sormat http://www.remotesensing.org/libtiff/libtiff.html
VTK	Visualization toolkit data IO (partial support) http://www.vtk.org

#### Additional notes about availability and quality assurance

The source code is manged in a public GIT repository [56], and the master branch of the version control system is normally kept in a stable state, i.e. before uploading changes it is tested whether all unit tests pass on the main developers platform (Linux x86_64). Bugs are tracked publicly [57], and for discussions public forums are available [58].

In addition, tagged releases of the source code are made available on the project home page. For these releases Debian GNU/Linux packages (*mia-tools* for end users, and *libmia-2.0-dev* for developers) are sponsored by the Debian-med project [59]. Back-ports to the current stable long time release version of Ubuntu Linux (12.04) are provided in a personal package archive [60].

Note, that while the GIT repository is not under an automatic test regime, the Debian GNU/Linux packaging process adds this additional layer of quality assurance because packaging only succeeds when all unit tests pass. Hence, when using the packages provided by the Debian/Ubuntu repositories it is ensured that all unit tests pass on the respective architecture.

#### Licensing considerations

MIA has been licensed under the terms of the GNU GPL version 3 for two reasons: Firstly, it is the software license that in our opinion best protects the interests of the software user, i.e. with its most prominent requirement that the distribution object code of a work covered by the GPL must be accompanied by the source code, or an offer to obtain the source code [61], the GPL license ensures that a user will always have access to the source code, the right to learn from it, tinker with it, improve it, contract someone to improve it, and redistribute it and derivative versions of it under the same GPL license. Secondly, MIA makes use of the GNU scientific library and IT++, which are both exclusively distributed under the terms of the GNU GPL, thereby imposing these terms also on the distribution of MIA. Note however, that software provided under the GPL can still be sold (cf. [62]), and it can also be distributed as part of medical devices where special certification may restrict the ability of the user to run changed code.

#### Overview over available algorithms and filters

At the time of this writing MIA version 2.0.10 is tagged as stable and it provides tools to run the following tasks on 2D and 3D images: 

• *image conversion*: combining series of 2D images to 3D images and extracting 2D slices from 3D images, selecting images from multi-frame data sets, and converting raw data to annotated 2D or 3D images, image file type conversion (implicitly based on filter output file type).

• *image filtering*: standard morphological filters (e. g. erode, dilate, thinning, open, close), pixel type conversion, various neighborhood filters in 2D and 3D (median, mean, Gaussian smoothing), point filters (binarize, invert, intensity-bandpass), segmentation filters (kmeans, seeded and basic watershed), and pipeline helper filters; in total 40 2D filters, 31 3D filters, and 9 FIFO filters are available. In addition, one FIFO *byslice* provides the means to make use of the available 2D filters in a FIFO pipeline.

• *image combination*: Combining pairs of images pixel-wise either arithmetically (subtract, add, multiply pixel values), or logically (and, or, xor,...).

• *image registration*: linear and non-linear registration in 2D and 3D optimizing various cost functions, and working on pairs or series of images. Non-linear registration includes transformations defined by B-splines [63] regularized by a vector-spline model [23], and dense vector fields regularized by a linear-elastic or a fluid-dynamical model [52,64], and possible optimizers are provided by the GNU Scientific library [65] and NLopt [66].

• *image segmentation*: 2D and 3D implementations for fuzzy c-means based segmentation [33,67] are provided.

• *Myocardial perfusion analysis*: A large set of tools is available that centers around motion compensation in perfusion imaging and its validation.

All filters, image similarity measures, optimizers, and most image combiners are implemented as plug-ins and are, hence, are also available through API calls. In addition to these image centric tools and filters, some facilities are available to create triangular meshes from 3D volume data and process these meshes.

An exhaustive, cross-referenced list of tools and plug-ins that are implemented within the current stable release is available on-line [28]. This documentation can also be created in the build process.

## Consent

Written informed consent was obtained from the patients whose data was used in use case one and three for the publication of this report and any accompanying images.

## Abbreviations

API: Application programming interface; CT, µCT: Computed tomography, micro computed tomography; ECG: Electrocardiogram; FIFO: First-in-first-out; FIFOF: First-in-first-out filter; GPL: GNU General public license; LV: Left ventricle; MRI: Magnetic resonance image; PET: Positron emission tomography; POSIX: Portable operating system interface; PPA: Personal package archive; RED: Rigid external distraction; RV: Right ventricle; STL: Standard template library; TDD: Test driven development.

## Competing interests

The authors declare that they have no competing interests.

## Authors’ contributions

GW is responsible for the software design and development, and for the main body of the article. PK and MJLC provided the data, medical expertise and planning for the motion compensation in myocardial perfusion analysis (1st use case). MS and JJH provided the data expertise and execution of analysis of tooth data (2nd use case). ThH provided the data, medical expertise and execution of analysis for the evaluation of the distraction osteogenesis treatment (3rd use case). All authors read and approved the manuscript.
